# Improved Mechanical Property Synergy of CoCrNiAlTi Medium-Entropy Alloy Through Boron Microalloying, Thermomechanical Treatment and Aging Treatment

**DOI:** 10.3390/ma19050871

**Published:** 2026-02-26

**Authors:** Po-Sung Chen, Huai-Te Wu, Hao Chen, Jason Shian-Ching Jang, I-Yu Tsao

**Affiliations:** 1 Institute of Materials Science and Engineering, National Central University, Taoyuan 320, Taiwan; 2 Department of Mechanical Engineering, National Central University, Taoyuan 320, Taiwan

**Keywords:** medium-entropy alloys, nonequiatomic ratio, microalloying, thermomechanical treatment

## Abstract

Medium-entropy alloys (MEAs) with a simple phase structure and nanoprecipitates have excellent mechanical properties and considerable potential for advanced structural applications. The current study investigated the effect of boron microalloying and thermomechanical treatment on the microstructure evolution and mechanical properties of Co_43_Cr_15_Ni_30_Al_5_Ti_7_ and (Co_43_Cr_15_Ni_30_Al_5_Ti_7_)_99.7_B_0.3_ MEAs. X-ray diffraction analysis revealed a single phase of face-centered cubic (FCC) structure in all as-cast samples. After cold rolling and recrystallization annealing were completed, a clear ordered FCC (L1_2_) phase was observed concurrently with the FCC matrix. In the alloy doped with 0.3 at.% B, the grain size was refined from 600 to 200 nm. TEM analysis revealed a nano-sized L1_2_ phase coherently embedded in the FCC matrix. Analysis of the mechanical properties of boron-doped MEA samples revealed that cold rolling to 80% thickness followed by annealing at 900 °C for 2 h and aging at 750 °C for 4 h yielded the best mechanical performance. Among all samples, the alloy doped with 0.3 at.% boron achieved an optimal combination of mechanical properties (yield strength: 1817 MPa; ultimate tensile strength: 2313 MPa; ductility: 14.5%).

## 1. Introduction

Metallic materials require ultrahigh specific strength and acceptable plasticity for most structural applications, such as aircraft landing gear, rocket cases, high-performance shafts and tubes, and high-strength fasteners [1,2]. Fine-grain strengthening is a conventional method that can increase tensile strength by up to 2.0 GPa through the fabrication of ultrafine grains measuring several dozen nanometers; however, this method sacrifices uniform ductility [3]. This effect results from the trade-off between ductility and strength, which is a universal law for structural materials. Therefore, the development of methods to achieve ultrahigh stress while maintaining acceptable uniform plasticity in materials with a face-centered cubic (FCC) structure remains challenging.

A novel design concept of multiprincipal element alloys (MPEAs)—high-entropy alloys (HEAs)—was proposed in 2004 to overcome the limitations of current alloy design concepts [4,5]. MPEAs and HEAs comprise a wide variety of alloy designs with unique characteristics, leading them to have improved properties relative to traditional alloys [6,7,8]. In addition to conventional equiatomic HEAs, nonequiatomic and medium-entropy alloys (MEAs) have been developed [9,10,11]. These alloys both retain the unique material properties of HEAs and increase the variety of the alloy design [12,13,14,15].

Researchers have recently developed Co-rich CoCrNi MEAs with more favorable mechanical properties than those of conventional MEAs [16,17,18] by tuning the compositions to reduce phase stability and stacking fault energy. CoCrNi MEAs have demonstrated exceptional mechanical properties due to the assistance of twinning-induced plasticity [16,17,18]. The critical stress threshold necessary to trigger the deformation twin is reached when the environmental temperature decreases to 77 K, at which point the deformation twin can simultaneously increase both yield strength and plasticity.

Precipitation strengthening has successfully been applied to increase the yield strength of single FCC-structured CoCrNi MEA through the addition of Al and Ti elements [19,20,21,22,23,24]. The outstanding mechanical performance of these MEAs at ambient temperature may be attributable to the joint effect of precipitation hardening from the coherently ordered FCC-structured nanoparticles and high work-hardening capability of the FCC matrix [19,20].

Grain refinement is among the most effective methods for further improving the mechanical properties of MEAs, particularly their ductility. Microalloying with a grain refiner, such as boron, considerably reduces the grain size, which in turn increases the area of grain boundaries, which can improve the alloy strength by accumulating dislocation density [25,26,27,28,29,30].

In this study, a Co_43_Cr_15_Ni_30_Al_5_Ti_7_ MEA with exceptional mechanical properties (>2 GPa yield strength and approximately 10% plasticity) was selected as the base alloy for studying the effect of grain refinement on as-cast alloy by microalloying with boron at first. Then the thermomechanical treatment (TMT) was conducted to further modify the microstructure and improve the mechanical property synergy of the boron-doped MEA.

## 2. Experimental Procedure

A series of (Co_43_Cr_15_Ni_30_Al_5_Ti_7_)_100-x_B_x_ MEAs (x = 0–0.4, labeled B0.0, B0.1, B0.2, B0.3, and B0.4 at.%) were prepared with a mixture of high-purity Co (99.9%), Cr (99.99%), Ni (99.99%), Al (99.99%), Ti (99.99%), and B (99.5%). The alloys were fabricated through arc melting under a high-purity argon atmosphere and the Ti-getter was also used to avoid oxidation. Each alloy was turned over four times for remelting to ensure homogeneity. Finally, the ingots were remelted and cast into a water-cooled copper mold with dimensions of 40 mm (L) × 20 mm (W) × 10 mm (T) through drop casting. Subsequently, the ingots were homogenized at 1000 °C for 6 h and then water-quenched.

The homogenized ingots were cold rolled to ensure 80% thickness reduction at room temperature. Subsequently, the deformed samples were annealed at 900 °C/s at soaking times of 2 h and then water-quenched. The recrystallized samples were aged at 750 °C at a soaking time of 4 h. MEA density was measured according to Archimedes’ method. The structure of the alloys was analyzed through X-ray diffraction (XRD; D2, Bruker, Billerica, MA, USA) involving Cu-Kα radiation. For metallograph observation, optical microscopy (OM; BX51M, Olympus, Tokyo, Japan), electron backscatter diffraction (EBSD; HKL Channel 5, Oxford Instruments, Hobro, Denmark), and transmission electron microscopy (TEM; JEM-2000FXII & JEM-2100, JEOL, Tokyo, Japan) were performed. For OM observation, the samples were polished in Al_2_O_3_ suspension liquid with particle sizes of 1 and 0.05 µm. For EBSD characterization, the samples were prepared through electropolishing. For TEM analysis, the samples were prepared through focused ion and electron beam microscopy (Versa 3D, FEI, Hillsboro, OR, USA).

A Vickers hardness tester (HV-115; Mitutoyo, Kawasaki, Japan) was performed to measure hardness under 5 kg loading for 10 s. Silicon carbide sandpaper was used to grind the samples from #80 to #2000 grit sizes. The tensile mechanical properties were tested with a universal testing machine (HT9102; Hung Ta, Taichung, Taiwan) under quasi-static loading with a strain rate of 1 × 10^−4^/s. The gauge dimensions of the tensile testing specimen were 5 mm (L) × 2 mm (W) × 1.5 mm (T).

## 3. Results and Discussion

On the basis of the specifications of previously reported Co_43_Cr_15_Ni_30_Al_5_Ti_7_ MEAs with ultrahigh strength, a series of B-microalloyed (Co_43_Cr_15_Ni_30_Al_5_Ti_7_)_100−x_B_x_ MEAs (Table 1) were prepared [24]. The configuration entropy of the alloys was within 11.18–11.35 kJ/mol. The entropy of the MEAs increased slightly as the amount of B doping increased.

**Figure 1:**
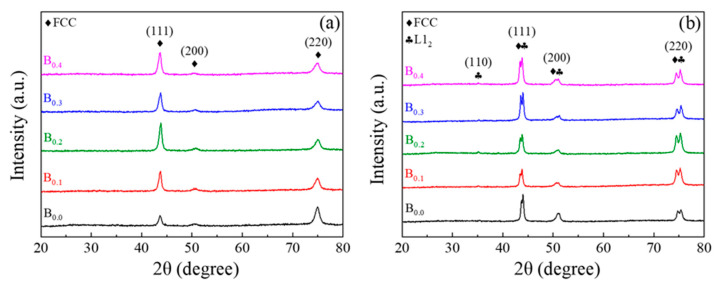
XRD spectra of (Co_43_Cr_15_Ni_30_Al_5_Ti_7_)_100-x_B_x_ MEAs (**a**) as-rolled and (**b**) as-aged.

XRD analysis indicated that after TMT processing, all MEA series exhibited two set of characteristic peaks: one FCC phase coexisting with another ordered FCC phase (also referred to as L1_2_ crystal structure), as illustrated in Figure 1. Additionally, because of the lattice distortion effect caused by the atomic size difference $\delta_{r}$ resulting from the doping of smaller B atoms, the diffraction peaks shifted slightly to higher diffraction angles for the alloys with more B added. The atomic size difference was defined as follows:

(1) $$\delta_{r}=100\sqrt{\sum_{i=1}^{n}c_{i}{\left(1-r_{i}/\bar{r}\right)}^{2}}$$

where $\bar{r}=\sum_{i=1}^{n}c_{i}r_{i}$, $c_{i}$ and $r_{i}$ denote the atomic concentration and atomic size of the *i*th element, respectively. Table 1 presents the atomic size differences and lattice constants of the MEAs, calculated by Debye–Scherrer method. Meanwhile, the atomic size differences $\delta_{r}$ of all studied alloys are about 5%, favoring the formation of a single solid solution structure [31].

To estimate the theoretical densities of the MEAs, the assumption of mixtures rule for a disordered solid solution was applied, as given in Equation (2):

(2) $$\rho =\frac{\sum C_{i}A_{i}}{\sum \frac{C_{i}A_{i}}{\rho_{i}}}$$

where *c_i_*, *A_i_*, and *ρ_i_* denote the atomic percentage, atomic weight, and density of the *i*th constituent element, respectively. The measured densities of all MEA series were similar at approximately 8.0 g/cm^3^, aligning with the theoretical densities calculated according to the mixing rule (Table 2).

**Figure 2:**
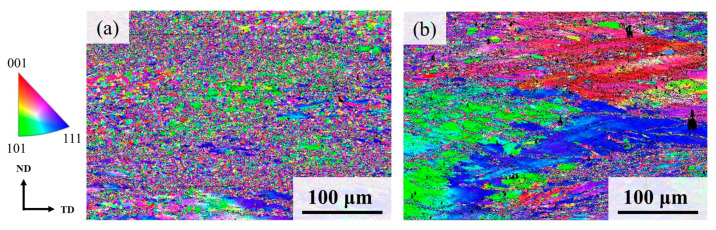
EBSD images of (**a**) B0.0 and (**b**) B0.3 MEAs after TMT processing (cold rolling to 80% thickness, annealing at 900 °C for 2 h, and aging at 750 °C for 4 h).

**Figure 3:**
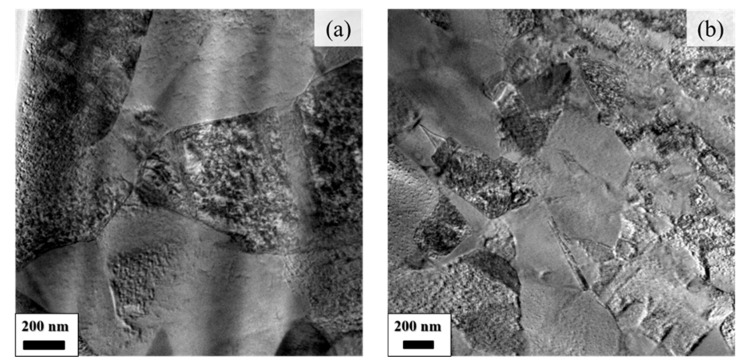
TEM bright field images of (**a**) B0.0 and (**b**) B0.3 MEAs after TMT processing (cold rolling to 80% thickness, annealing at 900 °C for 2 h, and aging at 750 °C for 4 h).

**Figure 4:**
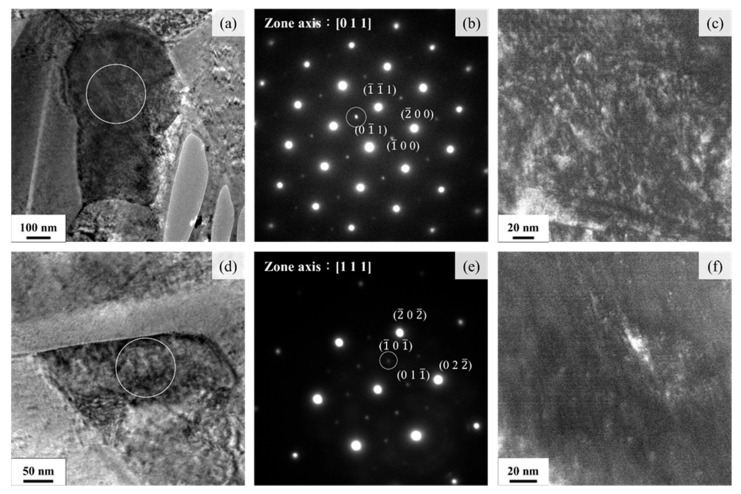
TEM images and nano diffraction patterns of B0.0 and B0.3 alloys. (**a**) Bright field image of B0.0 sample. (**b**) B [011] diffraction pattern of B0.0 sample. (**c**) Dark field image of B0.0 sample from (0 $\bar{1}$ 1) reflection of the L1_2_ phase. (**d**) Bright field image of B0.3 sample. (**e**) B [111] diffraction pattern of B0.3 sample. (**f**) Dark field image of B0.3 sample from ($\bar{1}$ 0 $\bar{1}$) reflection of the L1_2_ phase.

EBSD images of the B0.0 and B0.3 MEAs after cold rolling to ensure 80% thickness followed by annealing at 900 °C for 2 h and aging at 750 °C for 4 h revealed a nearly recrystallized microstructure composed of randomly oriented ultrafine grain structures in the B-free alloy (Figure 2). By contrast, only a small portion of the B0.3 alloy microstructure was recrystallized. The average grain size of both alloys after TMT processing was less than 1 μm, presenting difficulties to their resolution and estimation through EBSD. However, TEM characterization revealed that the grain size of the boron-doped alloy (B0.3, 359 ± 36 nm) was smaller than that of the boron-free alloy (B0.0, 678 ± 22 nm), as illustrated in Figure 3. This finding aligns with the expected outcome of grain refinement through B doping [28,29]. Moreover, the selected area diffraction pattern of both samples after TMT processing exhibited a clear superlattice corresponding to an ordered FCC phase, indicating the presence of the L1_2_ precipitate in the FCC matrix phase. The size of the L1_2_ precipitate was estimated to be approximately 15 nm for both the B0.0 and the B0.3 alloy, as indicated in Figure 4.

**Figure 5:**
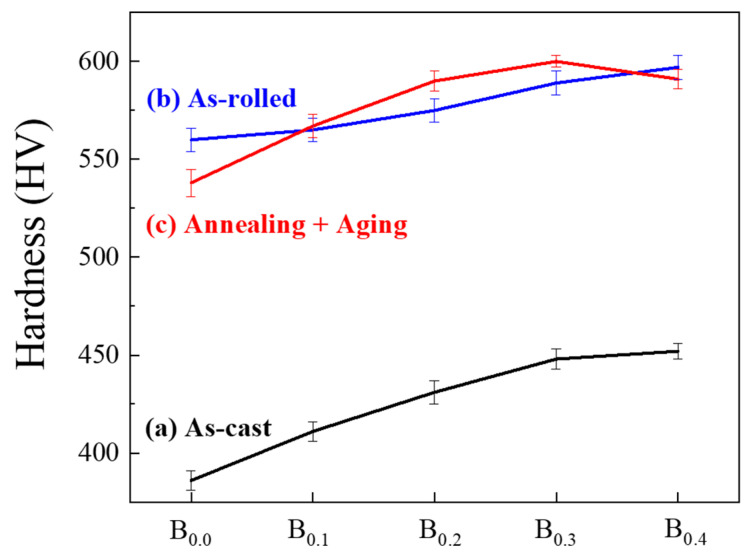
Hardness as a function of boron content in (Co_43_Cr_15_Ni_30_Al_5_Ti_7_)_100-x_B_x_ MEAs after (a) casting, (b) rolling, and (c) annealing and aging.

**Figure 6:**
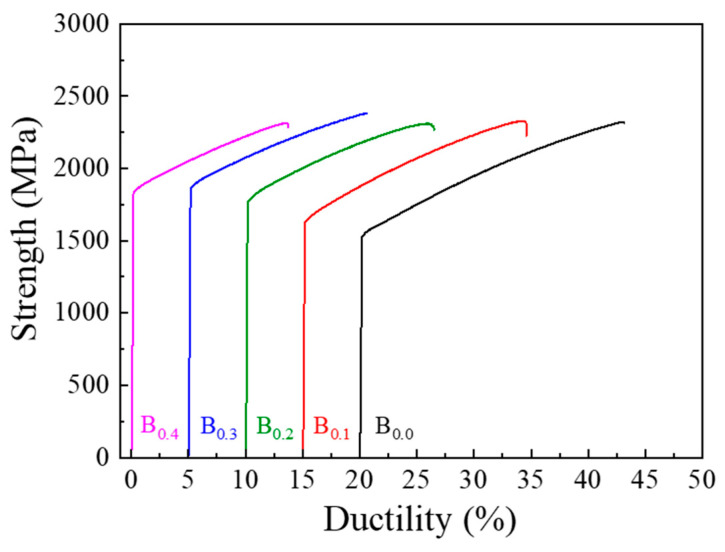
Mechanical tensile stress–strain curves of (Co_43_Cr_15_Ni_30_Al_5_Ti_7_)_100-x_B_x_ MEAs after TMT processing (cold rolling to 80% thickness, annealing at 900 °C for 2 h, and aging at 750 °C for 4 h).

Boron doping substantially increased alloy hardness following casting, rolling, annealing, and aging, as detailed in Table 3. Additionally, the results of tensile testing of these MEAs after TMT processing also present similar trend, as illustrated in Figure 5 and Table 3. Whereas the yield strength and ultimate tensile strength increased with greater boron content, ductility decreased with increasing boron content, as indicated in Figure 6 and Table 3. And the reason is considered that minor boron addition would reduce the grain size of alloy, thereby increasing the grain-boundary area and then improving the strength and hardness, consistent with Hall–Petch relationship [26]. Saturation appeared to emerge at a boron content of 0.3%, suggesting that excess boron addition beyond this threshold cannot provide additional alloy-strengthening benefits. The optimal combination of mechanical properties (yield strength: 1817 MPa, ultimate tensile strength: 2313, ductility: 14.5%) was also observed in the B0.3 alloy after cold rolling to 80% thickness, recrystallization at 900 °C for 2 h, and aging at 750 °C for 4 h. The yield strength of the B0.3 alloy was 18% higher than that of the B-free alloy that underwent the same processing sequence.

From the perspective of physical metallurgy, grain refinement can simultaneously improve both the yield strength and ductility of alloys. In this study, minor B doping successfully reduced the average grain size of the Co_43_Cr_15_Ni_30_Al_5_Ti_7_ base alloy. During TMT processing, interstitial B atoms reduce the interfacial free energy and inhibit the interfacial migration of nucleation sites, which obstruct the propagation of dislocations during recrystallization, leading to retardation in recrystallization behavior and reduced grain size at a given annealing temperature. The yield strength of the B0.0 and B0.3 MEAs increased to approximately 150 MPa after aging at 750 °C for 4 h, and the ductility of both alloys remained above 14.5%. This finding is attributable to the strengthening effect of the nano precipitate of the L1_2_ phase. The ordered and coherent FCC matrix lattice can effectively obstruct dislocation slipping, improving yield strength. Accordingly, among all MEA samples subjected to TMT processing in this study, B0.3 exhibited the best combination of mechanical properties, including 1817 MPa yield strength, 2313 MPa ultimate tensile strength, and 14.5% ductility.

## 4. Conclusions

On the basis of the experimental results, the microstructure evolution and mechanical properties of the fabricated (Co_43_Cr_15_Ni_30_Al_5_Ti_7_)_100−x_B_x_ MEAs (x = 0–0.4) can be summarized as follows:

1. XRD analysis indicated that after TMT processing, all MEA series exhibited two sets of characteristic peaks, indicating the coexistence of one FCC phase with another ordered FCC phase (also referred to as an L1_2_ crystal structure).
2. TEM characterization revealed that the boron-added alloy had a smaller grain size than did the boron-free alloy (B0.3, 359 ± 36 nm; B0.0, 678 ± 22 nm). This aligns with the expected outcome of grain refinement through B doping. The selected area diffraction pattern of the alloy samples after TMT processing exhibited a clear superlattice corresponding to an ordered FCC phase, indicating the coexistence of the L1_2_ precipitate in the FCC matrix phase. The size of the L1_2_ precipitate was estimated to be approximately 15 nm for both the B0.0 and the B0.3 alloy.
3. The hardness and tensile strength of the boron-doped alloys after TMT processing (80% cold rolling, recrystallization at 900 °C for 2 h, and aging at 750 °C for 4 h) increased with greater boron content and became saturated at a boron content of 0.3%. The B0.3 MEA possessed the optimal combination of yield strength (1817 MPa), ultimate tensile strength (2313 MPa), and ductility (14.5%). These findings indicate that through suitable TMT processing and boron doping, (Co_43_Cr_15_Ni_30_Al_5_Ti_7_)_99.7_B_0.3_ alloys with synergistic mechanical properties can be obtained.

## Figures and Tables

**Table 1:** Entropy, atomic size difference, and lattice constant of FCC matrix and L1_2_ phase of (Co_43_Cr_15_Ni_30_Al_5_Ti_7_)_100-x_B_x_ MEAs.

Alloy with B Content (at.%)	Entropy ΔS (KJ·mol^−1^)	Atomic Size Difference δr (%)	Lattice Constant (Å)	
			L1_2_ Phase	FCC Matrix
B0.0	11.179	5.128	3.588	3.566
B0.1	11.234	5.210	3.605	3.578
B0.2	11.277	5.290	3.609	3.574
B0.3	11.315	5.370	3.598	3.564
B0.4	11.351	5.448	3.609	3.574

**Table 2:** Theoretical and measured density of (Co_43_Cr_15_Ni_30_Al_5_Ti_7_)_100-x_B_x_ MEAs.

Alloy with B Content (at.%)	Theoretic Density (g/cm^3^)	Measured Density (g/cm^3^)	Error (%)
B0.0	8.028	8.045	0.20
B0.1	8.023	8.042	0.24
B0.2	8.017	8.016	0.01
B0.3	8.011	8.003	0.10
B0.4	8.005	7.999	0.07

**Table 3:** Tensile mechanical properties of (Co_43_Cr_15_Ni_30_Al_5_Ti_7_)_100-x_B_x_ MEAs after TMT processing (cold rolling to 80% thickness, annealing at 900 °C for 2 h, and aging at 750 °C for 4 h).

Composition	Hardness (Hv)	Yield Stress (MPa)	Ultimate Strength (MPa)	Ductility (%)
B0.0	538 ± 7	1534 ± 18	2328 ± 29	23.2 ± 0.4
B0.1	567 ± 6	1645 ± 27	2297 ± 42	19.1 ± 1.2
B0.2	590 ± 5	1756 ± 12	2260 ± 68	14.7 ± 1.9
B0.3	600 ± 3	1817 ± 34	2313 ± 67	14.5 ± 1.7
B0.4	591 ± 5	1760 ± 16	2293 ± 31	15.6 ± 0.6

## Data Availability

The original contributions presented in this study are included in the article. Further inquiries can be directed to the corresponding author.
